# Supplementary material for: Cooperative behaviors and social interactions in the carnivorous bat Vampyrum spectrum
Source: PLoS One. 2025 Aug 20;20(8):e0321338. doi: 10.1371/journal.pone.0321338 (PMC12367153; doi:10.1371/journal.pone.0321338)
Supplement: Attachment — Submitted filename: Review of PONE.docx [file pone.0321338.s012.docx]

Review of PONE-D-25-10917

Cooperative Behaviors and Social Interactions in the Carnivorous Bat Vampyrum spectrum

This is an interesting paper that provides innovative information about an interesting aspect of the biology of a bat species that has not been sufficiently studied. I welcome the opportunity to comment on the manuscript. I believe the paper is very sound, and no doubt it will be very attractive to PLoS ONE readership. I have some comments that should help drive the messages home:

Line 121 and following. It says “amidst typical dry tropical forest vegetation”. I doubt that there is such a thing as typical dry tropical forest. The dry tropical forest of Costa Rica is definitely different than the dry tropical forest of Honduras, Guatemala, and Mexico. In fact it is so different that the dry tropical forest in Costa Rica allows species that live only in wet tropical forest (such as V spectrum) to live there. Please add a brief description of the vegetation and I would love to see a brief discussion about why V spectrum lives in tropical rainforest always except in Costa Rica and a few areas in South America (I understand, though, that this is not a biogeography or an ecology study, so if you don´t wish to do that, just add the vegetation description I ask).

Please also add more details about the tree itself and its surroundings. Height? DBH? Is the canopy of this tree connected to others? Is this the tallest tree in the vicinity? Describe the hollow; for example, can it be accessed from the ground? Or is the entrance to the cavity up higher in the trunk? Dimensions of the entrance, height and diameter of the cavity (likely tapered, from how wide to how wide?) Is it a single cavity with one entrance only? How high are the V spectrum? The Saccopteryx? Is there a separation between them? How far apart?

Lines 128 and following. So it was originally four individuals and with the newborn five? Why is there no discussion of the other two individuals (assuming there were four and then five)? Sex? Please provide more detail as to the individual members of the group.

Line 145. The camera was regularly checked, how often?

Line 157. I rest my case. The reader cannot know where are the two bat species

Line 208. Only four prey were seen being carried in? That is odd for these bats. Did you collect any remains from under the bats? Or I guess it´s limitations of your video equipment.

Line 216. Says “may takes” should say “may take”

Lines 208 and following, can you tell us a bit about the prey species being eaten in all these videos? Itching!

227. Emitting vocalizations. Did you see some physical movement of the wings? In my experience both Chrotopterus and Vampyrum “beg” by vocalizing and also by shaking their wings noticeably. Sadly your videos do not catch the start of the interaction. In our experience, even before the adult carrying the prey shows in the camera field of view, the recipient (it can also be an adult!!) begins shaking its wings and vocalizing. You did not witness or record this?

228 says “the bat sharing prey” it would be more accurate to say “the bat providing”. In the end, the provider and the recipient are sharing the prey.

Overall the study is very interesting. It would help to have a bit more information about the roost itself and the authors should provide a clearer discussion of the bats that compose the colony. Was it four or five? Only two apparent adults? The other two were subadults plus the newborn? Nothing is clear there. I am sure once they fix these issues it will be a very fine paper.
