# Supplementary material for: Cooperative behaviors and social interactions in the carnivorous bat Vampyrum spectrum
Source: PLoS One. 2025 Aug 20;20(8):e0321338. doi: 10.1371/journal.pone.0321338 (PMC12367153; doi:10.1371/journal.pone.0321338)
Supplement: Attachment — Submitted filename: Response to Reviewers.docx [file pone.0321338.s014.docx]

**Comments Reviewer #1:**

**Original comment:** Line 76: You say “living in” twice

**Revised version:** Deletion of one “living in” (line 59)

**Original comment:** In the methods section “Individual distinction by size estimation” it wasn’t immediately clear why this analysis was being conducted. Only later, in the results, did it become apparent that it was used to differentiate younger and older individuals. I suggest clarifying the purpose of this analysis earlier in the methods section

**Revised version:** Added clarification to the original sentence: “Due to the absence of individual markings on the animals, we employed supplementary methods **for the distinction of individuals (adult vs. pup)** to support our observations and interpretations of behaviors, particularly in prey-sharing interactions”. (line 176)

**Original comment:** Line 216: Remove “may”

**Revised version:** Removed “may” and replaced it with “**is often**”. (line 206)

**Original comment:** Line 288: “our data suggest that…”

**Revised version:** Deletion of one “s” at the end: „our data suggest“ (line 282)

**Original comment:** Line 303: This sentence could be made clearer—perhaps: “…the approaching bat successfully wrestled the prey away from the original possessor.”

**Revised version:** Adoption of the proposed wording: Occasionally, conflicts over prey arose, as evidenced by one video depicting a tug-of-war scenario where **the approaching bat successfully** **wrestled the prey away from the original possessor.** (line 296-298)

**Original comment:** Discussion (Lines 356–368): After reading about biparental care, I found myself wondering whether you have any observations or data regarding the relative investment in pup care by the male and female. I understand that without tagging individuals this may be difficult, but if there is any indication—such as who provided prey items—it could be valuable to include here

**Revised version:** Addition of two new sentences for more supporting arguments:

**“The additional video recordings from 2024 that documented the presence of a newborn pup, revealed that nursing females frequently remained in the roost during the night with the young pup for approximately two months. During this period, the adult male was regularly observed bringing prey items into the roost and sharing them either with the older pup or with the female. Although the absence of individual identification precludes definitive assessments of the relative contributions of each parent, the available evidence suggests that mainly males bring in the prey item and then share it with another individual”.** (line 335-342)

**Comments Reviewer #2:**

**Original comment:** Line 121 and following. It says “amidst typical dry tropical forest vegetation”. I doubt that there is such a thing as typical dry tropical forest. The dry tropical forest of Costa Rica is definitely different than the dry tropical forest of Honduras, Guatemala, and Mexico. In fact it is so different that the dry tropical forest in Costa Rica allows species that live only in wet tropical forest (such as V spectrum) to live there. Please add a brief description of the vegetation and I would love to see a brief discussion about why V spectrum lives in tropical rainforest always except in Costa Rica and a few areas in South America (I understand, though, that this is not a biogeography or an ecology study, so if you don´t wish to do that, just add the vegetation description I ask).

**Revised version:** Clarification that we referred to the typical dry forest in Guanacaste, Costa Rica:

“The focal roost is located within a partially hollow yet living tree of the species *Manilkara chicle*, amidst typical dry forest vegetation **of Guanacaste, Costa Rica** and adjacent to a dried riverbed” (line 104).

Also, addition of a short description of the dry forest in this area: **“This area of tropical dry forest is characterized by a stratified vegetation structure comprising canopy, understory, shrub, and ground layers, each supporting distinct plant and animal communities. The canopy layer ranges from 20 to 30 meters in height and is composed of deciduous trees with broad crowns. The understory consists of trees reaching 10 to 20 meters, typically with light canopies and slender trunks. Beneath this, the shrub layer (2–5 m) is dominated by thorny, multi-stemmed plants.** (line 105-110)

**Author additional comment:** The study of Martínez-Fonseca et al. 2022 with *V. spectrum* was also conducted with a roost/a group situated in tropical lowland dry forest in Nicaragua.

**Original comment:** Please also add more details about the tree itself and its surroundings. Height? DBH? Is the canopy of this tree connected to others? Is this the tallest tree in the vicinity? Describe the hollow; for example, can it be accessed from the ground? Or is the entrance to the cavity up higher in the trunk? Dimensions of the entrance, height and diameter of the cavity (likely tapered, from how wide to how wide?) Is it a single cavity with one entrance only? How high are the V spectrum? The Saccopteryx? Is there a separation between them? How far apart?

**Revised version:** More details about the tree and roost: **The roost site is located within a mature tree reaching a height of approximately 20 meters, with a maximum diameter at breast height (DBH) of 1m. The tree forms part of a structurally interconnected canopy, although it is not the tallest individual in the immediate vicinity. The roost itself is situated within a hollow trunk, the entrance of which is spanned approximately 2 meters above ground level. The opening is roughly 80 cm wide at its base, progressively narrowing to approximately 50 cm with height, and lacks any secondary openings along the trunk.”** (line 110-116)

The *V. spectrum* bats occupy the highest section of the hollow tree, **positioned approximately 4 to 4.5 meters above the ground**, while a colony of 15 smaller bats, *Saccopteryx bilineata,* inhabits a lower section near the entrance of the tree hole, **around 3 meters above ground level, maintaining spatial separation from *Vampyrum spectrum*.** (line 120-123)

**Original comment:** Lines 128 and following. So it was originally four individuals and with the newborn five? Why is there no discussion of the other two individuals (assuming there were four and then five)? Sex? Please provide more detail as to the individual members of the group

**Revised version:** Addition of words/sentences for clarifying that there were always max. 4 bats present in the roost at the same time:

“3) additional video recordings in 2024 showed that **after the older pup/subadult left the colony a newly born pup was born soon after** and being nursed by its mother **and that the other large individual was male** (because the penis was visible). The newborn pup was identified as male from further video recordings in 2024. The group size never exceeded four individuals in the roost at the same time**.”** (line 128-132)

**Author additional comment:** The confusion may have arisen from the mentioning of additional video recordings from 2024, despite the primary data presented in the paper originating exclusively from 2023. The 2024 material was referenced solely as anecdotal material supporting information and is not part of the analysed dataset.

**Original comment:** Line 145. The camera was regularly checked, how often?

**Revised version:** Addition of the time indication: “Over the course of three months, the camera was regularly checked **and** cleaned **at least once a week**, **as well as** equipped with a new SD card, and fitted with fresh batteries before being reinstalled at the fixed location within the tree **when necessary**.” (line 144-147)

**Original comment:** Line 157. I rest my case. The reader cannot know where are the two bat species

**Revised version:** No direct changes in the text/ see revised parts above

**Original comment:** Line 208. Only four prey were seen being carried in? That is odd for these bats. Did you collect any remains from under the bats? Or I guess it´s limitations of your video equipment.

**Revised version:** Additional sentence to clarify: **The behavior classified here as “bringing prey into the roost” refers to video sequences in which a single bat was observed carrying prey into the cavity, but the subsequent interaction—whether the bat consumed the prey itself or shared it with another individual—could not be determined** (line 217-220)**.**

**Author additional comment:** This limitation is most likely due to the restricted duration of the recordings, which were limited to one-minute clips.

Prey-provision behavior was recorded on 12 occasions; however, it is likely that this behavior occurred more frequently than documented, as it may have gone unrecorded due to several limitations of the camera setup.

During each visit to the roost, we collected all animal remains found on the ground, which primarily consisted of bird feathers and, occasionally, small mammal bones. These samples are currently undergoing analysis and will be presented in a future publication, with the aim of providing more detailed insights into the dietary composition of this group.

**Original comment:** Line 216. Says “may takes” should say “may take”

**Revised version:** Grammar correction: word “take” now without the “s”. (line 225)

**Original comment:** Lines 208 and following, can you tell us a bit about the prey species being eaten in all these videos? Itching!

**Revised version:** Additional short sentence regarding the prey species: “**Prey items were identified as either small mammals (rats or mice) or birds.**” (line 223/224)

**Author additional comment:** Species level identification of prey will hopefully achieve as soon as the collected regularly remains are fully analysed. The video quality forbid further identification other than “small mammal” or “bird”.

**Original comment:** 227. Emitting vocalizations. Did you see some physical movement of the wings? In my experience both Chrotopterus and Vampyrum “beg” by vocalizing and also by shaking their wings noticeably. Sadly your videos do not catch the start of the interaction. In our experience, even before the adult carrying the prey shows in the camera field of view, the recipient (it can also be an adult!!) begins shaking its wings and vocalizing. You did not witness or record this?

**Revised version:** No direct changes in the text

**Author additional comment:** Additional video recordings captured further instances of prey-sharing, including the initiation of the interaction. However, our footage does not reveal clear wing movements associated with prey transfer. Instead, we observed whole-body movements by the receiving bat as it approached the individual carrying the prey. This may be attributable to the spatial constraints of the roost, which is relatively confined, as well as the low position of the entrance. Incoming bats must crawl upward toward the main roosting area while holding prey in their mouths, potentially limiting the expression of more conspicuous behavioral gestures.

We agree that the recipient bat can also be an adult (as mentioned in the discussion part line 330- 338) and in at least two videos from 2024 it seems to be the case that one adult individual (male) is sharing with another adult individual (female), especially in times when the female is nursing a young pup like it was the case in nov/dec 2024.

Originally it was planned to catch the animals from this roost during the dry season in 2024 and non-inversive mark them but mainly due to bad weather conditions it was not possible for us. We will sure try again in the upcoming season end of 2025.

With a song meter bat mini all vocalisations from the bats during the night were separately recorded during these 3 months. We are currently analysing the vocalizations. We are hoping to put together the prey-sharing behavior with the respective vocalisations that are typically emitted in this context. This will be the topic of a further publication.

**Original comment:** 228 says “the bat sharing prey” it would be more accurate to say “the bat providing”. In the end, the provider and the recipient are sharing the prey

**Revised version:** Changed the term “prey sharing” to “prey provision” for better clarification (in all cases throughout the text)

**Author additional comment:** We agree that “prey provision” is more accurate in this context because none of the recorded videos show actual joint consumption of the prey item. Rather, the behavior appears to involve a full transfer of the prey from one individual to another. In all observable cases (except the tug-of war-video), the prey-carrying bat hands over the entire prey item without retaining any portion for itself. This interpretation is supported by the visible chewing behavior: the receiving bat begins chewing the prey, while the transferring individual does not engage in feeding and typically proceeds to rejoin the others at the main roosting site.
